# Should females prefer old males?

**DOI:** 10.1002/evl3.250

**Published:** 2021-08-24

**Authors:** Julia Carolina Segami, Martin I. Lind, Anna Qvarnström

**Affiliations:** ^1^ Department of Ecology and Genetics, Animal Ecology Uppsala University Uppsala SE‐75236 Sweden

**Keywords:** Ageing, direct benefits, extra‐pair mating, female preference, genetic benefits, germline senescence, mate choice, reproductive senescence

## Abstract

Whether females should prefer to mate with old males is controversial. Old males may sire offspring of low quality because of an aging germline, but their proven ability to reach an old age can also be an excellent indicator of superior genetic quality, especially in natural populations. These genetic effects are, however, hard to study in nature, because they are often confounded with direct benefits offered by old males to the female, such as experience and high territory quality. We, therefore, used naturally occurring extra‐pair young to disentangle different aspects of male age on female fitness in a natural population of collared flycatchers because any difference between within‐ and extra‐pair young within a nest should be caused by paternal genetic effects only. Based on 18 years of long‐term data, we found that females paired with older males as social partners experienced an overall reproductive advantage. However, offspring sired by old males were of lower quality as compared to their extra‐pair half‐siblings, whereas the opposite was found in nests attended by young males. These results imply a negative genetic effect of old paternal age, given that extra‐pair males are competitive middle‐age males. Thus, offspring may benefit from being sired by young males but raised by old males, to maximize both genetic and direct effects. Our results show that direct and genetic benefits from pairing with old males may act in opposing directions and that the quality of the germline may deteriorate before other signs of senescence become obvious.

**Impact summary**

Male age may influence the number and quality of the offspring he produces. This is due to senescence, the gradual deterioration of organism function with age, which can affect the germline cells quality (i.e., genetic or indirect parental effects) and the ability to provide resources (i.e., direct parental effects). However, proven ability to survive until old age may mean that older fathers instead will produce high‐genetic‐quality offspring, able to survive, and get old themselves. Additionally, old males may also be able to provide their offspring with better or more resources due to gained experience. These reproductive pros and cons of old male partners impose a dilemma for mate choosing females. Should they avoid or prefer old males to ensure direct and indirect benefits? This question has not yet been fully answered because direct and indirect effects of paternal age are hard to separate in the wild. We circumvented this problem by comparing nestlings sired by different males of different ages within the same brood. Therefore, we were able to distinguish the indirect (genetic) from direct (environmental) effects of biological fathers, as the environment and maternal genes were constant. We used 18 years of long‐term data on a collared flycatcher population of which 812 individuals were genotyped to assign paternity. We found that direct benefits increase with male age, but at the same time the germline quality decreases. Moreover, this deterioration happens before other signs of senescence become evident. These findings have profound consequences for the general understanding of the evolution of mate choice, which is rarely considered in the context of senescence. Our study is one of very few exploring the effects of male senescence in the wild and it is the first one to disentangle genetic from direct benefits in the context of mate choice.

Ageing is the physiological deterioration of organism functionwith age and is widespreadin nature (Shefferson et al. 2017). Increasing age can affect fitness of the individual (Lemaître and Gaillard 2017) and also its offspring through negative parental age affects (Monaghan et al. 2020), either because of reduced general phenotypic performance or because of accumulation of deleterious germline mutations. Therefore, age could influence partner choice and females may avoid mating with old males to minimize these negative effects. However, there are also numerous positive effects associated with increased age. In fact, females are often assumed to prefer to mate with older males to gain access to an experienced mate with superior resources and proved ability to survive (Grant and Grant 1987; Conner 1989; Côté and Hunte 1993; Takagi 2003; Dupont et al. 2018). How these negative and positive aspects of male age translate into number and quality of the offspring they sire and/or raise and hence influence the fitness of females that have chosen to breed with them remain open questions. Whether females should prefer to mate and pair with old males has therefore become a controversial subject (Brooks and Kemp 2001; Griffith et al. 2002; Beck and Promislow 2007; Dean et al. 2010; Lifjeld et al. 2011; Dupont et al. 2018; Rodríguez‐Muñoz et al. 2019). Accordingly, there is a need to investigate whether detrimental effects of old age are common in males and also to examine to what extent such effects are strong enough to compromise the reproductive success of females in the wild (Lemaître and Gaillard 2017).

Traditionally, natural selection was thought to favor females that prefer older males because such males should be more experienced and thereby also more likely to have access to high‐quality resources and to provide better parental care, which would provide direct benefits to the female (Grant and Grant 1987; Conner 1989; Côté and Hunte 1993; Takagi 2003; Dupont et al. 2018). Moreover, mating with older males can also give indirect (genetic) benefits to the female in terms of more fit offspring. The “good genes” hypothesis predicts a preference for older individuals based on the logic that survival until older ages requires high‐quality genes, which in turn will be passed on to the offspring (Trivers 1972; Kokko and Lindstrom 1996; Kokko 1998; Bouwman et al. 2007). In accordance with these expectations, there are several studies demonstrating that older males are more likely to attract females for mating (Conner 1989; Côté and Hunte 1993), to become socially paired (Weatherhead 1984; Alatalo et al. 1986), to be less likely to lose paternity in the broods they attend (Michálková et al. 2019), and to be more likely to gain extra pair paternity (Dickinson 2001; Bouwman et al. 2007; Cleasby and Nakagawa 2012; Michálková et al. 2019).

Possible negative aspects of pairing with relatively old males can be both in terms of reduced phenotypic skills (i.e., reduced resource holding potential or ability to provide parental care) as a consequence of senescence of the soma, but also genetically in terms of mutation accumulation in the male germline. Physiological senescence with increasing age is widespread across organisms (Shefferson et al. 2017), and examples from birds that could result in direct costs are reduced to foraging efficiency and nest defense (Newton and Rothery 2002; Bouwhuis and Vedder 2017). In contrast, purely genetic costs to the offspring of old fathers stayed for a long time undetected in wild animals. This contrasts to laboratory studies, where offspring from old male mice were found to have decreased reproduction and longevity (García‐Palomares et al. 2009), and negative paternal age effects on offspring life span have also been found in *Drosophila* (Priest et al. 2002) and captive populations of zebra finch (Noguera et al. 2018). Moreover, in humans, offspring life span decreases with increasing age of the father (Gavrilov and Gavrilova 1997; Kemkes‐Grottenthaler 2004). Such negative effects of male age were for a long time considered irrelevant in the context of evolution of mate choice in nature. Predation and other sources of mortality were thought to remove individuals from the population before the onset of senescence (Medawar 1951; Comfort 2011)⁠. Moreover, even if a few males would survive until the onset of senescence the likelihood of mating with them would be minimal meaning that selection on females to avoid mating with too old males should be negligible (Finch 1998). The few males that do show senescence may also compensate by being more experienced at acquiring resources or providing parental care further lowering the potential gain of avoiding these males as mates. However, these arguments have recently become questioned.

Because the reproductive success of males is very much determined by the ability to secure a mate and sire as many offspring as possible, males are expected to invest in secondary sexual characters, expensive behaviors (such as male‐male interactions, courtship, territory defense), and/or sperm competition, perhaps even at the expense of their germline maintenance (Lemaître and Gaillard 2017). Germline cells deteriorate with advanced age (Kong et al. 2012)⁠ and mutation accumulation in the germline cells can have negative effects on the quality of the offspring in natural populations (Pizzari et al. 2008; Velando et al. 2011)⁠. Mutation rates have also been shown to be higher in males than in females (Kong et al. 2012; Smeds et al. 2016)⁠ probably due to the large number of cell divisions during spermatogenesis. Moreover, recent findings in *Drosophila* suggest that the fitness effects of negative mutations often increase nonlinearly with age (Brengdahl et al. 2020). Thus, it is possible that effects of male senescence on offspring number and quality, especially in the form of mutation accumulation in the male germline, have been overlooked in natural populations. There is now growing evidence that various effects of senescence may be observed also in natural populations (Bonduriansky and Brassil 2002; Nussey et al. 2013; Bouwhuis and Vedder 2017; Froy et al. 2019; Gaillard and Lemaître 2020)⁠, which suggests that negative effects of advanced age can be important for mating decisions, at least if senescent males are present in the population or if the germline deteriorates before other visible signs of ageing occur. Still, relatively few studies have investigated possible effects of male reproductive senescence in the wild (Lemaître and Gaillard 2017), with a study on house sparrows being a rare exception. This study focused solely on possible genetic effects by using a cross‐fostering design and found that offspring of old parents had lower reproductive success, but with a sex‐specific effect such that old paternal age only influenced the fitness of sons and old maternal age only affected the fitness of daughters (Schroeder et al. 2015).

Taken together, the decision to mate with an old male reflects a balance between positive direct (Grant and Grant 1987; Conner 1989; Côté and Hunte 1993; Takagi 2003; Dupont et al. 2018) and genetic (Trivers 1972; Kokko and Lindstrom 1996; Bouwman et al. 2007) benefits of choosing an old mate, but also potential costs associated with mating with too old males that experience senescence of the soma (Bouwhuis and Vedder 2017) or germline (Pizzari et al. 2008; Velando et al. 2011; Kong et al. 2012), which are outlined in Figure 1. How these different aspects of male age translate into number and quality of the offspring they sire and/or raise and hence influence the fitness of females that have chosen to breed with them remain open questions. The relative importance of these different effects has to our knowledge not previously been disentangled in studies on natural populations. Our study aims to fill this knowledge gap using two approaches: (1) using long‐term breeding data of collared flycatchers (*Ficedula albicollis*) to assess the effect of paternal age on offspring fledge number and offspring recruitment and (2) by using naturally occurring extra‐pair offspring to disentangle direct and genetic effects of paternal age on offspring quality while keeping maternal genetic effects constant. Collared flycatchers are small passerine birds that preferably breed in nest boxes which allows long term monitoring. The flycatcher population of Öland has been monitored over 18 years and pedigrees as well as age records are available since 2002 (Qvarnström et al. 2010)⁠. We used these long‐term breeding data to test whether females benefit from breeding with older males. In addition, we also genotyped a large number of offspring to specifically single out possible genetic effects associated with paternal age such as increased genetic quality of offspring with male age due to the male's proven ability to survive and/or decreased genetic quality of offspring with male age due to germline deterioration. In this case, we only have information of the age of the social male as the extra‐pair male is unknown. Nevertheless, we can infer the relative age of the extra‐pair male based on two facts. First, most extra‐pair young (EPY) are known to be sired by competitive middle‐aged males as revelated by other studies (Dickinson 2001; Bouwman et al. 2007; Cleasby and Nakagawa 2012; Michálková et al. 2019). Second, even if females would randomly mate with extra‐pair males the age structure of the studied population indicates that young males breeding for their first time would be cuckolded by equally old or older males (i.e., on average older males). Similarly, males belonging to the oldest age classes should be cuckolded by younger males.

**Figure 1:**
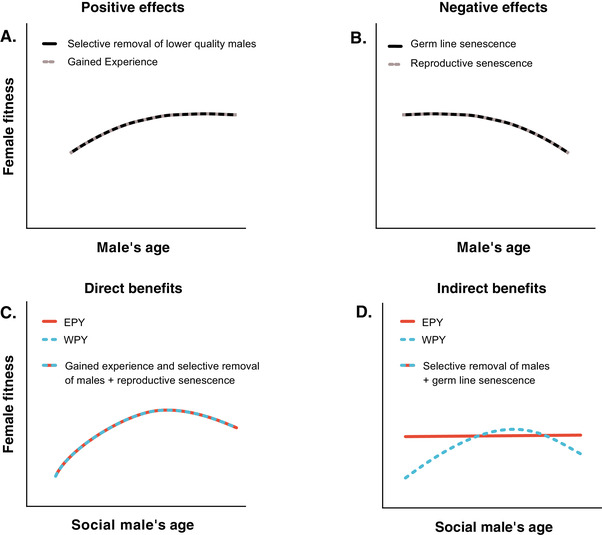
The first two panels show expected relationships between female fitness in terms of number and quality of offspring produced and the age of her mate through several possible processes. The second two panels illustrate how naturally occurring extra pair young can be used to disentangle effects mediated by resources or genes provided by the males. This is because these offspring share the same nest environment, including resources provided by the social male, and maternal genes but are sired by males of (on average) different ages. (A) Positive effects of male age on female fitness. Increased male experience and/or selective removal of males of low quality may lead to higher quality of the resources such as territories and paternal care that are provided by the older males. Selective removal of low‐quality males may also ensure genetic benefits to females selecting older males that have proven their ability to survive. The expected net benefits to females with increasing male age will level off as selection will have removed the males of lowest quality and due to an expected less sharp learning curve late in life. (B) Negative effects of male age on female fitness. Reproductive senescence may negatively affect the phenotypic quality of males toward the end or their lives, leading to middle‐aged males being of highest phenotypic quality and therefore providing the best direct benefits to their females. Old males may also experience decreased germline quality as deleterious mutations accumulate with male age leading to a decline in genetic benefits obtained by females selecting older males as mates. These negative fitness effects are expected to accelerate in association with “tipping points” when a decline in male general phenotypic quality, for example, makes them unable to defend high‐quality territories. (C) Direct material benefits from socially pairing with males of different ages will have similar effects on the number and phenotypic quality of within‐ (WPY) and extra‐pair young (EPY). (D) Assuming that extra‐pair young are mainly sired by middle‐aged competitive males, we expect EPY to be of superior genetic quality as compared to WPY when the social male is very young or very old. This is because very young social males have not yet proven their ability to survive to older ages (i.e., selection has not yet removed low‐quality males) and very old social males may experience germline senescence.

## Methods

### DATA COLLECTION

The collared flycatcher (*Ficedula albicollis*) is a small migratory passerine of the family *Muscicapidae*; they overwinter in sub‐Saharan Africa and breed in Europe (Qvarnström, Rice, and Ellegren 2010). At the beginning of the reproductive season in early May, males arrive to the breeding grounds and establish territories that they advertise to females. Once a female starts building the nest, the male defends its territory from other males and predators. After laying eggs, the female starts incubating and the male provides her food. When the eggs hatch, both the male and the female share the task of feeding the nestlings and guarding the nest. They are insectivorous with a preference for caterpillar larvae as food for their offspring (Shirihai and Svensson 2018). The population breeding on Öland (57°100N, 16°580E) has been monitored in deciduous and mixed forests with over 2000 nest boxes across the island during the reproductive season between May and July since 2002 (Qvarnström, Rice, and Ellegren 2010). Metal rings with a unique identity code are placed on every bird and morphological measurements as well as a blood samples are taken every year. Additionally, the offspring are weighted (±0.1g) at day 6 and day 12 before fledgling with a spring Pesola of 30 g. This long‐term data collection allowed us to determine social pedigrees, age, and fitness measurements. In addition, we used a subset of breeding events to specifically test for possible effects of paternal age on the genetic quality of the offspring. We genotyped 812 offspring from 154 nests. These nests are from the following years: 2002, 2004–2005, 2008, and 2010–2016.

To assess extra‐pair and within‐pair paternity of social couples offspring, we compared 12 microsatellite loci (FhU1, FhU2, FhU3, FhU4, Fhy223, Fhy301, Fhy304, Fh401, Fhy403, Fhy407, Fhy454, and PdoU5) of the offspring with their known mother and their social father using the software *Cervus 3.0.3* (Marshall et al. 1998; Kalinowski et al. 2007) as previously described in similar studies (Alund et al. 2013, 2018). For this analysis, data were simulated for 10,000 offspring with five candidate fathers assuming the sampling of 70% of the population (Jones et al. 2010). Only individuals where at least six microsatellite loci were compared were included and the confidence level used to establish extra pair paternity in the pairwise comparison between offspring and social father was >95%. It was not possible to identify the extra‐pair sires.

### STATISTICAL ANALYSIS

Our overall strategy for statistical analyses was to construct a limited number of biologically relevant models to test the hypotheses outlined in Figure 1. These models are presented in Table S1. In general, these models include both a linear and quadratic effect of male age, because nonlinear fitness effects of age may be present for both direct (Forslund and Pärt 1995) and genetic effects (Fig. 1D) (Pizzari et al. 2008). These linear and nonlinear fitness effects are also expected to interact with predictors of interests. For offspring recruitment probability, we expect that the effect of male survival to next year can interact with age and age^2^ due to expected age‐dependent solutions to life‐history trade‐offs (Clutton‐Brock 1984). We also expect that the effect of EPY status on offspring mass can differ depending upon parental age, in both a linear and nonlinear fashion. Moreover, although reproductive data are often underdispersed and arguments have been raised to analyze this type of data using flexible generalized linear models (Brooks et al. 2019), other arguments favor the robustness and interpretability of Gaussian models for this type of data (Knief and Forstmeier 2020). We therefore analyzed reproductive data using both methods, and because our results are robust to the method used, we present the Gaussian models in the Results section and the corresponding generalized linear models in the Supporting Information.

#### Long‐term breeding dataset

To understand the age structure of the population, data of individuals of known age (i.e., ringed the year of birth or as 1‐year old when plumage reveals exact age of the male) between 2002 and 2018 were visually explored with a population pyramid plot and age percentages of different age classes were calculated.

We analyzed the effect of the social fathers age on the number of fledged offspring (i.e., offspring who left the nest alive) and number of recruits (i.e., offspring of the given clutch that returned after migration to breed in the population on subsequent years), using both cross‐sectional data and longitudinal data of 1‐year‐old individuals to test for selection between age class 1 and 2. These males (*n* = 1094) were monitored between 2002 and 2018, and the total number of broods is 1527 of which 117 had 0 fledglings. All experimental (manipulated) broods in the study area were excluded for these analyses. We constructed separate linear mixed‐effect models with fledgling number or number of recruits as response variables. We fitted the age of the social father as explanatory variable and also age^2^, to investigate any nonlinear effect of age. For that purpose, age was mean centered before calculating age^2^. Male ID and year were included in the models as random effects on the intercept. Because both number of fledgling and number of recruits are count data that are generally underdispersed, we used both Gaussian distribution of the package *lme4* (Bates et al. 2015) and the “genpois” family distribution available in the *glmmTMB* package (Brooks et al. 2017). Because we got consistent results with both distributions, the Gaussian distribution models are presented in the *Results* section and the correspondent models fitted with *glmmTMB* can be found in the Supporting Information.

To test whether recruitment does not only depend on age but also on the survival of the parent to the next age category, we constructed a generalized mixed‐effect model with binomial distribution. Offspring recruitment (fail, success) was fit as response variable and age, age^2^, and male survival to the next year as explanatory variables, including all interactions. Male ID and year were included as random effects.

To determine whether mass at time of fledge is a good predictor for recruitment in our population, we constructed a generalized linear mixed‐effect model with binomial distribution having successful or unsuccessful recruitment as a response variable. Mass at day 12 (briefly before fledgling) and lay date were fitted as explanatory variables, whereas nest ID and year were fitted as random effects.

#### Extra‐pair offspring dataset

To determine the effect of the age of the social father on the offspring's weight at fledgling, we constructed a linear mixed‐effect model with the weight of the offspring at day 12 as the response variable. As explanatory variables we included the paternity status (EPY or within‐pair young [WPY]) and the number of nestlings in the nest, because there is evidence that this is an important factor influencing nestling weight (Källander and Smith 1990). We also fitted father's age and age^2^. Year and male ID were included as random effects on the intercept, male ID was then removed as it did not explain any variation in the model and caused singularity problems to the Gaussian model (male ID was retained in the Generalized model [Table S14], where it did not cause singularity issues). To avoid pseudo replication because individuals from the same brood can share the same father, we included a random slope of brood ID over paternity status. All statistical analyses were conducted in R 3.5.3 (R Core Team 2019), mixed‐effect Gaussian and binomial models were implemented using the package *lme4* (Bates et al. 2015), and Poisson models were implemented using the package *glmmTMB* (Brooks et al. 2017). All plots were created with the package ggplot2 (Wickham 2016) and show the last age category grouped when and additional category would yield less than 5% of all the data points. Significance was assessed using confidence intervals that were obtained using the *confint* function with Wald method.

## Results

The population shows a pyramidal age structure, where females and males show a similar age distribution with the majority of the population being composed by birds of 1 (45.8%) and 2 (25.5%) years old (Fig. S1; Table S2). Every age category experiences an approximate 50% reduction and thus, we find very few individuals of age 4 (8.5%) or older (5.3%). These older individuals are approximately 6.5% of the male population and 4% of the female population with the oldest individual registered of 9 years old for males and 8 years old for females.

### PATERNAL AGE AND OVERALL REPRODUCTIVE PERFORMANCE

**Figure 2:**
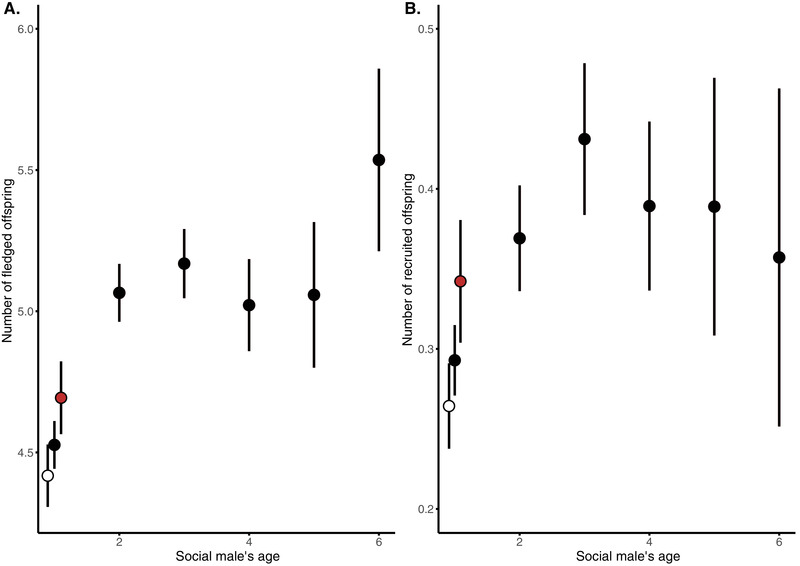
The number of fledglings (A) and recruits back into the breeding population (B) as a function of the social male's age in collared flycatchers. Symbols represent mean ± SE of the number of fledged offspring depending on the social male's age. Red symbol indicates data on males of age 1 that also survived to year 2, therefore controlling for selective disappearance. The white symbol indicates data on the males of age 1 who did not survive to year 4. There is a significant positive relationship between male age and reproductive performance that levels off with increasing age, but there is no apparent evidence for negative effects of male senescence on reproductive output when genetic and material effects are entangled.

Direct effects of the age of the social partner influence the number and quality of offspring that a female produces in the same way regardless of whether these offspring are WPY or EPY. Based on gained experience and selective removal of males with poor phenotypic quality (Fig. 1A), we expected females to experience higher reproductive performance when breeding together with older males. We found that females paired with older males as their social partners produced more (0.25 ± 0.06, *t* = 4.51, *P* < 0.001; Table S3; Fig. 2A) fledged offspring and more recruits that returned to the breeding population as adults themselves (0.06 ± 0.02, *t* = 3.53, *P* < 0.001; Table S5; Fig. 2B). In addition to the linear effect of the social fathers age on the number of fledglings, a significant quadratic effect of age captures the curvature of the response, where the positive effect of increased age of the social father weakens after age 2 (Table S3; Fig. 2). The increased benefit from breeding with older males was hence mainly driven by a lower performance of females paired with 1‐year‐old males that were breeding for the first time (Fig. 2). This increased performance observed between the first and second year of breeding in the cross‐sectional data can be driven either by increased performance of males due to gained experience or by selective removal of males of relatively poor quality. To disentangle these two possibilities, we therefore re‐analyzed the dataset using only 1‐year‐old individuals that were known to survive until their second year of breeding or after. Both fledglings and recruitment models lose the significant effect of age^2^, the linear effect of age is only borderline significant (depending upon model), and importantly the parameter estimate of age is much closer to zero in the longitudinal dataset (Tables S7 and S9). These results imply that selective removal of low‐quality individuals between the first and second breeding attempts plays an important role for explaining the benefits associated with selecting older males as breeding partners (Fig. 2).

**Figure 3:**
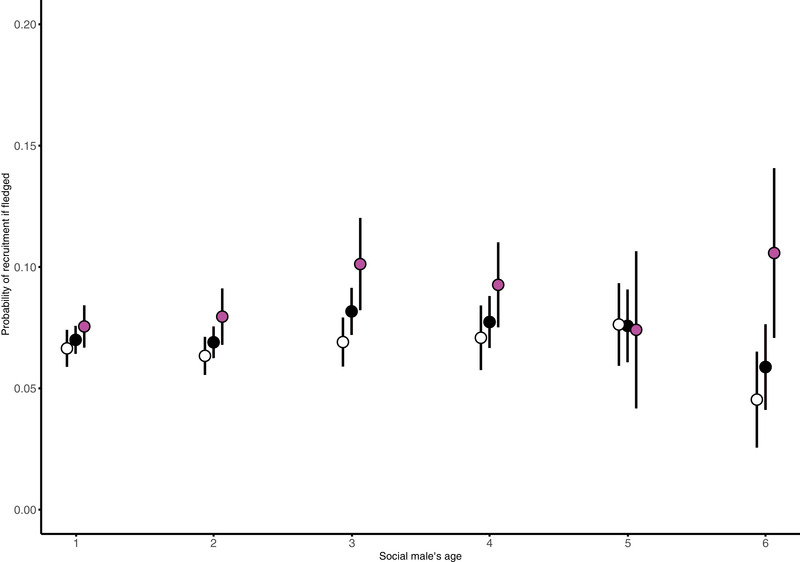
Probability of recruitment given successful fledgling depending on social male's age and survival to the next year. White symbols represent the mean probability ± SE of recruitment of offspring raised by males that died, magenta dots represent the mean probability ± SE of recruitment of offspring raised by males that survived, and black dots represent the mean ± SE of both. Offspring are more likely to recruit to the breeding population when they have fledged from nests attended by older males and males that survived to the following year themselves.

In addition, we also analyzed the probability that a fledged offspring would recruit into the breeding population. For our a priori model, which included a linear as well as quadratic effect of age and all interactions, we found no relationship between male survival to next year and offspring recruitment probability after fledgling (Table S13). However, if we allow for model simplification using AIC, the best model (which excludes interactions) showed that offspring in the nest of older males (−0.13 ± 0.05, *Z* = –2.49, *P* = 0.013; Table S12; Fig. 3) and males who survive to the next year (−0.21 ± 0.1, *Z* = −2.18, *P* = 0.03; Table S12; Fig. 3) have a higher recruitment probability.

### PATERNAL AGE AND GENETIC CONTRIBUTION TO OFFSPRING CONDITION

**Figure 4:**
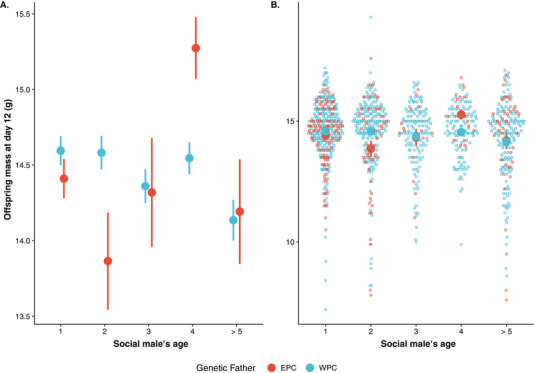
Mass at day 12 of extra‐pair (EPY, red color) and within‐pair (WPY, blue color) offspring depending on the social male's age. Within‐pair offspring are relatively heavier when the social male is young, but the opposite pattern with relatively heavier extra‐pair young is found when the social male is old. Offspring of males of age 5 or older are grouped in the plot but not on the analysis. (A) Mean ± SE of each age group. (B) Visualization of raw data points (with mean ± SE superimposed on top).

To estimate offspring quality, we used mass at day 12, which significantly predicts recruitment back to the breeding population (0.268 ± 0.026, *Z* = 10.116, *P* < 0.001, Table S11). We used 812 offspring from 154 social nests for which both the social male's age and paternity in the brood were known to disentangle possible effects of paternal age on the genetic quality of the offspring from direct effects of paternal age. We found a significant interaction between EPY status and age^2^ (Table 1) on offspring mass (condition). The interaction between EPY status and age should be interpreted as a more or less negligible linear effect on age for both EPY (nonsignificant weakly negative) and WPY (estimated to be flat), whereas the interaction between EPY status and age^2^ highlights fundamental differences in the nonlinear response, where EPY has a strong convex relationship between age and offspring mass with a minimum in young age and increased mass both for age 1 and especially age 4. In contrast, the quadratic relationship is convex and closer to zero for WPY. These interactions are illustrated in Figure 4, showing that WPY have a relatively higher mass than EPY in nests attended by 1‐ and especially 2‐year‐old males. In nests attended by very old males (4 year or older), we find that WPY have a relatively lower mass than EPY (Table 1; Fig. 4). Because competitive middle‐aged males mainly sire the EPY, both these results imply a negative genetic effect of paternal age on offspring mass as predicted based on age‐dependent germline deterioration (Fig. 1D).

**Table 1:** Linear mixed‐effect model with offspring mass at day 12 as a responsevariable

	Estimate	SE	*t*	*P*	CI 2.5%–97.5%
Intercept	14.217	0.548	25.926	**<0.001**	**13.12–15.30**
Age	−0.168	0.177	−0.951	0.344	−0.52–0.18
Paternity status (WPY)	0.671	0.210	3.194	**<0.002**	**0.25–1.09**
(Age)^2^	0.347	0.157	2.212	**0.03**	**0.04–0.66**
Total offspring in nest	−0.046	0.074	−0.626	0.532	−0.19–0.10
Age: Paternity status (WPY)	0.188	0.129	1.450	0.152	−0.08–0.44
Paternity status (WPY): (Age)^2^	−0.40	0.136	−2.921	**0.004**	**−0.67–0.13**

Random effects	σ	CI 2.5% –97.5%	*N* groups
Nest ID (intercept)	1.168	0.96–1.42	154
Paternity status	0.687 corr −0.54	–0.74 to −0.22	–
Year (intercept)	0.597	0.39 to 0.99	11
Residual	0.824	0.78 to 0.87	–

The explanatory variables are Age of the social father (Age), Paternity status (either within‐pair offspring [WPY] or extra‐pair offspring [EPY]), and total number of offspring in the nest. As random effects, we have Year and a random slope for Paternity status on Nest ID. Number of observations: 812. Number of broods: 154. A confidence interval that does not overlap with 0 indicates significance for the value in this case the Estimate. And in the P column all values of *p* <0.05 are considered significant and hence they are in bold. *P* <0.05 in all cases matches the significance given by the calculation of confidence intervals.

## Discussion

We found that female collared flycatchers benefit from breeding with old males, and especially should avoid 1‐year‐old males as social partners, to ensure overall high reproductive output. There was no evidence of reproductive male senescence lowering the direct, material benefits for females pairing with very old males. However, a comparison between WPY and EPY within a clutch revealed evidence consistent with deterioration of the germline of old males. Extra‐pair offspring had higher weight than within‐pair offspring in nest attended by old social males. Because old males are rare in the population, we assume that the EPY in these nests were sired by on average younger males. This result therefore demonstrates a decoupling of direct (material) and indirect (genetic) benefits of pairing with old males. Thus, female collared flycatchers could maximize their fitness by having their offspring sired by young males but raised by old males, where the direct benefits have an overall stronger effect on female fitness. Below we discuss these findings in more detail together with possible constraints on optimal female mating strategies.

The decision to mate with a male of a particular age reflects a balance of both direct and indirect benefits and costs associated with male age. Direct (or material) benefits are associated with resource provisioning and nest defense, and older males can often provide more resources due to increased experience and access to higher quality territories (Grant and Grant 1987; Conner 1989; Forslund and Pärt 1995; Takagi 2003). Indirect effects are instead related to the genetic effects of choosing a particular male, in terms of increased genetic quality of the offspring (Kempenaers and Dhondt 1993; Jennions and Petrie 2000; Griffith et al. 2002). Direct and genetic benefits are hard to disentangle in the wild, because both effects contribute to reproductive success of the social male, but we achieved this goal by using naturally occurring extra‐pair offspring. Any difference in performance between half‐sib WPY and EPY in a nest should be caused by genetic effects only, because males are not able to recognize its genetic offspring and therefore do not provide differential paternal care (Kempenaers and Sheldon 1996).

Firstly, we investigated the direct effect of paternal age. We found that females should prefer older males as social partners, and specifically avoid choosing young males as social partners. Both the number of fledglings and recruits (offspring returning to the breeding populations) increased with male age, which agree with a number of studies finding improved reproductive performance with age in birds (reviewed in Forslund and Pärt 1995). A significant nonlinear decline in the positive effect of male age suggested that the result was, as expected, mainly driven by low performance of 1‐year‐old males. Several mechanisms can underlie such patterns, such as increased experience (a direct effect), but also selective disappearance of low‐condition individuals, as well as age‐assortative mating (Zhang et al. 2015; Bouwhuis and Vedder 2017). By analyzing a longitudinal dataset with only individuals that survived at least 2 years, we found the effect of paternal age on the number of hatchlings and recruits almost disappeared, therefore selective disappearance of year‐1 males rather than increased experience is causing the increased reproductive performance of females having older males as their social partners. This result also excludes age‐assortative mating (where males and females of the same age preferentially breed together) as a possible explanation, because males surviving more than 1 year had a relatively high reproductive performance already as young. Moreover, we also found that the probability that a fledgling would recruit back into the breeding population increased not only with social male age but also with its survival to the following year. This suggests a good‐genes effect may be present for overwintering survival, further arguing for the importance of breeding with old males with proven ability to survive. Thus, the overall reproductive performance of females is clearly boosted by avoiding first‐year breeding males as social mates because selective disappearance ensures a generally higher quality of older males.

Although reproductive performance often increases with paternal age in birds (Forslund and Pärt 1995; Rebke et al. 2010; Torres et al. 2011; Auld et al. 2013; Zhang et al. 2015), few studies have separated the role of increased experience from selective disappearance of low‐performing males, and only in long‐lived birds. Selective disappearance contributed to the increased reproductive performance with age in blue‐footed booby (Torres et al. 2011) and mute swans (Auld et al. 2013), but had only a small effect in the common tern (Rebke et al. 2010; Zhang et al. 2015). For short‐lived birds, the role of selective disappearance has not been investigated in relation to male performance, but selective disappearance of low‐quality females is important in great tits (Bouwhuis et al. 2009) and collared flycatchers (Evans et al. 2011). There is thus a need for further studies aimed at separating the role of selective disappearance and increased experience for male reproductive performance, especially in short‐lived species.

We did not observe a decline in the reproductive performance of very old males (Figs. 2 and 3) as would have been expected based on reproductive senescence and a general decline in phenotypic performance and the ability to provide resources at very old ages (Fig. 1). This result contrasts against the observed strong decline in the reproductive performance of female collared flycatchers late in life (Gustafsson and Pärt 1990) and with evidence for senescence from other natural populations (Bonduriansky and Brassil 2002; Nussey et al. 2013; Bouwhuis and Vedder 2017; Froy et al. 2019; Gaillard and Lemaître 2020). Most of the previous scientific works have focused on females making it premature to make the conclusion that actuarial senescence is generally more evident in females even if this indeed appears to be the case in collared flycatchers. However, it has been suggested that sex‐specific reproductive strategies may result in sexual selection favoring males that invest heavily in keeping a competitive phenotype in shape also at old ages and perhaps even at the expense of their germline maintenance (Lemaître and Gaillard 2017). Effects of germline senescence are difficult to assess, because genetic effects generally are masked by direct effects of the breeding environment in natural populations (but see below).

Secondly, we assessed the indirect effect of paternal age. Because selective removal of poor‐quality 1‐year‐old individuals resulted in increased reproductive success, we expected that the females would also get genetic benefits from choosing old males. However, there may also be a genetic cost associated with choosing old males due to germline senescence (Fig. 1B). To separate direct and genetic effects, we used naturally occurring extra‐pair paternity to investigate the relative difference in mass between WPY and EPY within a clutch. This approach has previously been used to investigate if females gain genetic benefits from extra‐pair mating (e.g., Kempenaers et al. 1992; Foerster et al. 2003; Charmantier et al. 2004), but has to our knowledge never been used to investigate effects of male germline senescence. The shared nest environment means that both EPY and WPY benefit from being raised by old males. Because most males in the population are young (Fig. S1), but middle‐aged males are most likely to get extra‐pair paternity (Dickinson 2001; Bouwman et al. 2007; Cleasby and Nakagawa 2012; Michálková et al. 2019), we expect that EPY in nest with young males as social partners are sired by older males, whereas EPY in nests with very old males as social partners are sired by relatively younger, competitive middle‐aged males, because very old males are rare in the population. The half‐siblings (i.e., within‐ and between‐pair young) sharing the same nest are exposed to the same direct effects of paternal age (Fig. 1C), which in the flycatcher case lack evidence for a negative effect of actuarial male senescence in broods raised by very old males (Figs. 2 and 3). If females gain genetic benefits by mating with old males that have proven their ability to survive (Fig. 1A), we expect that EPY in nests attended by young males should be of better condition (have higher mass) than WPY (Kempenaers et al. 1992; Fig. 1D). For nests attended by very old males, we also expected EPY to be of better condition (have higher mass), but in this case due to germline deterioration among these very old social males (Fig. 1D). As expected, EPY indeed outperformed WPY in nests attended by very old males. Surprisingly, however, we found evidence for negative performance effects associated with paternal age also in nests attended by young males where within‐pair offspring outperformed EPY that probably had been sired by “middle‐aged” competitive males. These results imply that the germline of these competitive males may deteriorate before other signs of senescence become obvious. This finding is consistent with the idea that sexual selection has led to the evolution of males that prioritize somatic maintenance at the expense of their germline maintenance (Lemaître and Gaillard 2017). One should, however, note that the negative effect was stronger for nests attended by 2‐year‐old rather than 1‐year‐old males. A possible explanation to this finding is that that the two sources of genetic benefits (i.e., avoidance of the effects of germline deterioration by having offspring sired by young males vs. ensuring the effects of good genes by having offspring sired by old males with proven ability to survive) even each other out in nests attended by 1‐year‐old males.

Because mating outside the social pair bond is associated with obvious costs to females, such as exposure to sexually transmitted disease (Sheldon 1993) or reduced parental care by the social mate (Arnqvist and Kirkpatrick 2005; Griffin et al. 2013), much previous scientific work has been allocated toward finding possible adaptive explanations to the wide spread occurrence of active female extra‐pair mating in birds. The most commonly proposed adaptive explanation is that females, who generally are constraint in their choice of social male, can obtain genetic benefits from extra‐pair mating (e.g., Jennions and Petrie 2000; Westneat and Stewart 2003; Akçay and Roughgarden 2007). That old males, with proven ability to survive and more attractive traits, often gain more extra‐pair matings as compared to other males is generally interpreted as evidence in support of this assumption (Akçay and Roughgarden 2007; Cleasby and Nakagawa 2012). However, this interpretation has recently become questioned (Brooks and Kemp 2001; Griffith et al. 2002; Beck and Promislow 2007; Dean et al. 2010; Lifjeld et al. 2011; Dupont et al. 2018; Rodríguez‐Muñoz et al. 2019) and some studies have even found evidence suggesting that EPY may be of lower genetic quality as compared to WPY (Sardell et al. 2011; Hsu et al. 2014). Thus, the overall evidence for large genetic benefits of extra‐pair mating is limited (Forstmeier et al. 2014). Our study implies that there may be negative genetic consequences of extra‐pair mating with old males. Specifically, our results are consistent with a deteriorating germline in old males. There are several mechanisms that can underlie deteriorating of the germline. Mutation accumulation is thought to be a major cause (Kong et al. 2012), but possibly also telomere shortening (Noguera et al. 2018) as well as passive epigenetic effects (Schroeder et al. 2015). We did not investigate the mechanism, but note that they all are evolutionary very similar, resulting from accumulation of deleterious effects with age because of weakened selection (Hamilton 1966).

Negative parental age effects are seen as a hallmark of ageing, and several laboratory studies have found that offspring of old parents have shorter life span (Lansing 1947; Rockstein 1957; Tracey 1958; O'Brian 1961; Kiritani and Kimura 1967; Gavrilov and Gavrilova 1997; Priest et al. 2002; García‐Palomares et al. 2009; Lind et al. 2015) and/or reduced fecundity (Priest et al. 2002; García‐Palomares et al. 2009). Although most often studied in females, negative paternal age effects have also been found in males of captive zebra finch (Noguera et al. 2018), *Drosophila* (Priest et al. 2002), and mice (García‐Palomares et al. 2009). However, although negative paternal age effects are found in the lab, they are much harder to detect in nature, where they can be masked by positive direct effects of increasing paternal age. Moreover, even negative effects on reproduction with age could reflect somatic ageing and diminished direct effects (Newton and Rothery 2002) and hide indirect (genetic) effects. To our knowledge, only one previous study has investigated genetic paternal age effect in wild birds, and found reduced lifetime reproduction of male offspring from old fathers in the house sparrow (Schroeder et al. 2015). The study on house sparrows did not consider the relative effects of direct (material) and indirect (genetic) parental age effects on offspring performance, which is the aim of our study. Instead, it focused on paternal genetic effects by using a cross‐foster design and did not investigate the effect of paternal genetic contribution to offspring performance against the same maternal genetic background. By using naturally occurring extra‐pair offspring, we can place our findings in relation to direct benefits from mating with older males and we are testing paternal genetic effects against the same maternal genetic background (i.e., by comparing halfsiblings). Although we found that within‐pair offspring of old social males are smaller than extra‐pair offspring in the same brood, WPY produced by these old males actually have a similar fledging weight as compared to WPY produced by younger males. This finding suggests that old males compensate the disadvantages of the deteriorated germline with increased parental investment. Because the reproductive success of males is very much determined by the ability to attract a mate (Andersson 1994), males are expected to invest heavily in secondary sexual characters, perhaps even at the expense of their germline maintenance (Lemaître and Gaillard 2017). Thus, males should prioritize keeping their soma in shape. In line with this, male ornamentation in collared flycatchers shows no signs of senescence, but is rather increasing with age (Evans et al. 2011), and Houbara bustards that invest considerably in sexual display also show rapid senescence of spermatogenic function (Preston et al. 2011). If males prioritize keeping their soma in shape, females may prefer old males to ensure direct benefits in terms of high‐quality territories and parental care.

Our findings also reveal that the ideal situation for a nestling is to be sired by a young male but to be raised in the nest attended by an old male. As a result, despite evident germline ageing, females should prefer old males as social partners because of the direct benefits but obtain extra‐pair mating with younger males. However, older males are generally dominant in aggressive interactions with other males (Andersson 1994) suggesting that young males may be prevented from successfully courting and mating with females socially paired with older males. Moreover, young males often arrive later in the season at the breeding grounds as compared to old males reducing the window when they may seek extra‐pair mating before the females lay all their eggs (Canal et al. 2012; Edme et al. 2016). Finally, males may respond to perceived lost paternity by depressed paternal investment, for example, in terms of reduced paternal care of offspring by their social mate, resulting in selection against EPC behavior in females (Arnqvist and Kirkpatrick 2005). Thus, both male‐male interactions and social males’ responses to perceived lost paternity are likely to constrain optimal female mating strategies in relation to male age.

To conclude, we used a combination of long‐term breeding data and naturally occurring EPY to disentangle the different aspects of male age on female fitness in a natural population of collared flycatchers. We found that females paired with older males experienced an overall reproductive advantage due to increased phenotypic quality of males belonging to the older age categories. Selective disappearance of low‐quality males was found to be a major driver behind the advantage of choosing old males as breeding partners. There was moreover evidence for a good gene benefit of choosing old males because males who survived were also more likely to have offspring recruiting back to the breeding population. However, we also found evidence for a negative genetic effect of mating with old males. Within the same nest, offspring sired by old males were of relatively lower quality as compared to their EPY half‐siblings, whereas the opposite was found in nests attended by young males. Direct and indirect benefits from pairing with old males hence act in opposing directions. Female flycatchers gain direct benefits from pairing with old males but genetic benefits from having young males siring their offspring.

## Supporting information

Figure S1. Age structure of the population since 2002 until 2018.
Figure S2. Age structure of the population per years since (A) 2008 until (I) 2016.
Table S1. A priori structure of the main models.
Table S2. Age structure total known age individuals monitored between 2002 and 2018.
Table S3. Linear mixed‐effect model (Gaussian) with fledgling number as response variable (cross‐sectional dataset).
Table S4. Generalized mixed‐effect model (genpois) with fledgling number as response variable (cross‐sectional dataset).
Table S5. Linear mixed‐effect model (Gaussian) with number of recruits as response variable (cross‐sectional dataset).
Table S6. Generalized mixed‐effect model (genpois) with number of recruits as response variable (cross‐sectional dataset).
Table S7. Linear mixed‐effect model with fledgling number as response variable (longitudinal dataset).
Table S8. Generalized mixed‐effect model (genpois) with fledgling number as response variable (longitudinal dataset).
Table S9. Linear mixed‐effect model (Gaussian) with number of recruits as response variable of longitudinal dataset (not including birds that died in year 1).
Table S10. Generalized mixed‐effect model (genpois) with number of recruits as response variable of longitudinal dataset (not including birds that died in year 1).
Table S11. Generalized mixed‐effect model (binomial) with recruitment as a response variable.
Table S 12. Generalized mixed‐effect model with binomial distribution for the response variable Recruitment (yes, no) with male's age and male's survival as explanatory variables.
Table S 13. Generalized mixed‐effect model with binomial distribution for the response variable Recruitment (yes, no) with male's age and male's survival as explanatory variables.

## References

[evl3250-bib-0001] Akçay, E., and J. Roughgarden. 2007. Extra‐pair paternity in birds: review of the genetic benefits. Evol. Ecol. Res. 9:855–868. https://repository.upenn.edu/biology_papers/12

[evl3250-bib-0002] Alatalo, R. V., L. Gustafsson, and A. Lundberg. 1986. Do females prefer older males in polygynous bird species? Am. Nat. 127:241–245.

[evl3250-bib-0003] Alund, M., S. Immler, A. M. Rice, and A. Qvarnstrom. 2013. Low fertility of wild hybrid male flycatchers despite recent divergence. Biol. Lett. 9 20130169. 23576780 10.1098/rsbl.2013.0169 PMC3645050

[evl3250-bib-0004] Alund, M., S. P. Schmiterlöw, S. E. McFarlane, and A. Qvarnström. 2018. Optimal sperm length for high siring success depends on forehead patch size in collared flycatchers. Behav. Ecol. 29:1436–1443.

[evl3250-bib-0005] Andersson, M. B. 1994. Sexual selection. Princeton Univ. Press, Princeton, NJ.

[evl3250-bib-0006] Arnqvist, G., and M. Kirkpatrick. 2005. The evolution of infidelity in socially monogamous passerines: the strength of direct and indirect selection on extrapair copulation behavior in females. Am. Nat. 165:S26–S37. 15795859 10.1086/429350

[evl3250-bib-0007] Auld, J. R., C. M. Perrins, and A. Charmantier. 2013. Who wears the pants in a mute swan pair? Deciphering the effects of male and female age and identity on breeding success. J. Anim. Ecol. 82:826–835. 23356697 10.1111/1365-2656.12043

[evl3250-bib-0008] Bates, D., M. Mächler, B. M. Bolker, and S. C. Walker. 2015. Fitting linear mixed‐effects models using lme4. J. Stat. Softw. 67:1–48.

[evl3250-bib-0009] Beck, C. W., and D. E. L. Promislow. 2007. Evolution of female preference for younger males. PLoS ONE 2 e939. 17895980 10.1371/journal.pone.0000939 PMC1976549

[evl3250-bib-0010] Bonduriansky, R., and C. E. Brassil. 2002. Rapid and costly ageing in wild male flies. Nature 420:377. 12459773 10.1038/420377a

[evl3250-bib-0011] Bouwhuis, S., and O. Vedder. 2017. Avian escape artists? Patterns, processes and costs of senescence in wild birds. In R. P. Shefferson, O. R. Jones, & R. Salguero‐Gomez (Eds.), The evolution of senescence in the tree of life (pp. 156–174). Cambridge Univ. Press, Cambridge, U.K.

[evl3250-bib-0012] Bouwhuis, S., B. C. Sheldon, S. Verhulst, and A. Charmantier. 2009. Great tits growing old: selective disappearance and the partitioning of senescence to stages within the breeding cycle. Proc. R. Soc. B Biol. Sci. 276:2769–2777. 10.1098/rspb.2009.0457 PMC2839957 19403537

[evl3250-bib-0013] Bouwman, K. M., R. E. van Dijk, J. J. Wijmenga, and J. Komdeur. 2007. Older male reed buntings are more successful at gaining extrapair fertilizations. Anim. Behav. 73:15–27.

[evl3250-bib-0014] Brengdahl, M. I., C. M. Kimber, P. Elias, J. Thompson, and U. Friberg. 2020. Deleterious mutations show increasing negative effects with age in Drosophila melanogaster. BMC Biol. 18:128. 32993647 10.1186/s12915-020-00858-5 PMC7526172

[evl3250-bib-0015] Brooks, M. E., K. Kristensen, K. J. van Benthem, A. Magnusson, C. W. Berg, A. Nielsen, et al. 2017. glmmTMB balances speed and flexibility among packages for zero‐inflated generalized linear mixed modeling. R J. 9:378–400.

[evl3250-bib-0016] Brooks, M. E., K. Kristensen, M. R. Darrigo, P. Rubim, M. Uriarte, E. Bruna, et al. 2019. Statistical modeling of patterns in annual reproductive rates. Ecology 100 e02706. 30916779 10.1002/ecy.2706

[evl3250-bib-0017] Brooks, R., and D. J. Kemp. 2001. Can older males deliver the good genes? Trends Ecol. Evol. 16:308–313. 11369109 10.1016/s0169-5347(01)02147-4

[evl3250-bib-0018] Canal, D., R. Jovani, and J. Potti. 2012. Male decisions or female accessibility? Spatiotemporal patterns of extra pair paternity in a songbird. Behav. Ecol. 23:1146–1153.

[evl3250-bib-0019] Charmantier, A., J. Blondel, P. Perret, and M. M. Lambrechts. 2004. Do extra‐pair paternities provide genetic benefits for female blue tits *Parus caeruleus*? J. Avian Biol. 35:524–532.

[evl3250-bib-0020] Cleasby, I. R., and S. Nakagawa. 2012. The influence of male age on within‐pair and extra‐pair paternity in passerines. Ibis 154:318–324.

[evl3250-bib-0021] Clutton‐Brock, T. H. 1984. Reproductive effort and terminal investment in iteroparous animals. Am. Nat. 123:212–229.

[evl3250-bib-0022] Comfort, A. 2011. The biology of senescence. Rinehart, New York.

[evl3250-bib-0023] Conner, J. 1989. Older males have higher insemination success in a beetle. Anim. Behav. 38:503–509.

[evl3250-bib-0024] Côté, I. M., and W. Hunte. 1993. Female redlip blennies prefer older males. Anim. Behav. 46:203–205.

[evl3250-bib-0025] Dean, R., C. K. Cornwallis, H. Løvlie, K. Worley, D. S. Richardson, and T. Pizzari. 2010. Male reproductive senescence causes potential for sexual conflict over mating. Curr. Biol. 20:1192–1196. 20579882 10.1016/j.cub.2010.04.059

[evl3250-bib-0026] Dickinson, J. 2001. Extrapair copulations in western bluebirds (*Sialia mexicana*): female receptivity favors older males. Behav. Ecol. Sociobiol. 50:423–429.

[evl3250-bib-0027] Dupont, S. M., C. Barbraud, O. Chastel, K. Delord, S. Ruault, H. Weimerskirch, et al. 2018. Young parents produce offspring with short telomeres: a study in a long‐lived bird, the Black‐browed Albatross (*Thalassarche melanophrys*). PLoS ONE 13 e0193526. 29561856 10.1371/journal.pone.0193526 PMC5862442

[evl3250-bib-0028] Edme, A., P. Munclinger, and M. Krist. 2016. Female collared flycatchers choose neighbouring and older extra‐pair partners from the pool of males around their nests. J. Avian Biol. 47:552–562.

[evl3250-bib-0029] Evans, S. R., L. Gustafsson, and B. C. Sheldon. 2011. Divergent patterns of age‐dependence in ornamental and reproductive traits in the collared flycatcher. Evolution 65:1623–1636. 21644953 10.1111/j.1558-5646.2011.01253.x

[evl3250-bib-0030] Finch, C. E. 1998. Variations in senescence and longevity include the possibility of negligible senescence. J. Gerontol. A Biol. Sci. Med. Sci. 53:B235–B239. 18314551 10.1093/gerona/53a.4.b235

[evl3250-bib-0031] Foerster, K., K. Delhey, A. Johnsen, J. T. Lifjeld, and B. Kempenaers. 2003. Females increase offspring heterozygosity and fitness through extra‐pair matings. Nature 425:714–717. 14562103 10.1038/nature01969

[evl3250-bib-0032] Forslund, P., and T. Pärt. 1995. Age and reproduction in birds — hypotheses and tests. Trends Ecol. Evol. 10:374–378. 21237076 10.1016/s0169-5347(00)89141-7

[evl3250-bib-0033] Forstmeier, W., S. Nakagawa, S. C. Griffith, and B. Kempenaers. 2014. Female extra‐pair mating: adaptation or genetic constraint? Trends Ecol. Evol. 29:456–464. 24909948 10.1016/j.tree.2014.05.005

[evl3250-bib-0034] Froy, H., A. M. Sparks, K. Watt, R. Sinclair, F. Bach, J. G. Pilkington, et al. 2019. Senescence in immunity against helminth parasites predicts adult mortality in a wild mammal. Science 365:1296–1298. 31604239 10.1126/science.aaw5822

[evl3250-bib-0035] Gaillard, J. M., and J. F. Lemaître. 2020. An integrative view of senescence in nature. Funct. Ecol. 34:4–16.

[evl3250-bib-0036] García‐Palomares, S., S. Navarro, J. F. Pertusa, C. Hermenegildo, M. A. García‐Pérez, F. Rausell, et al. 2009. Delayed fatherhood in mice decreases reproductive fitness and longevity of offspring. Biol. Reprod. 80:343–349. 18923156 10.1095/biolreprod.108.073395

[evl3250-bib-0037] Gavrilov, L. A., and N. S. Gavrilova. 1997. Parental age at conception and offspring longevity. Rev. Clin. Gerontol. 7:5–12.

[evl3250-bib-0038] Grant, B. R., and P. R. Grant. 1987. Mate choice in Darwin's finches. Biol. J. Linn. Soc. 32:247–270.

[evl3250-bib-0039] Griffin, A. S., S. H. Alonzo, and C. K. Cornwallis. 2013. Why do cuckolded males provide paternal care? PLoS Biol. 11 e1001520. 23555193 10.1371/journal.pbio.1001520 PMC3608547

[evl3250-bib-0040] Griffith, S. C., I. P. F. Owens, and K. A. Thuman. 2002. Extra pair paternity in birds: a review of interspecific variation and adaptive function. Mol. Ecol. 11:2195–2212. 12406233 10.1046/j.1365-294x.2002.01613.x

[evl3250-bib-0091] Gustafsson, L., and T. Pärt. 1990. Acceleration of senescence in the collared flycatcher Ficedula albicollis by reproductive costs. Nature 347(*6290*):279–281. 10.1038/347279a0

[evl3250-bib-0041] Hamilton, W. D. 1966. The moulding of senescence by natural selection. J. Theor. Biol. 12:12–45. 6015424 10.1016/0022-5193(66)90184-6

[evl3250-bib-0042] Hsu, Y. H., J. Schroeder, I. Winney, T. Burke, and S. Nakagawa. 2014. Costly infidelity: low lifetime fitness of extra‐pair offspring in a passerine bird. Evolution 68:2873–2884. 24931726 10.1111/evo.12475 PMC4303991

[evl3250-bib-0043] Jennions, M. D., and M. Petrie. 2000. Why do females mate multiply? A review of the genetic benefits. Biol. Rev. 75:21–64. 10740892 10.1017/s0006323199005423

[evl3250-bib-0090] Jones, A. G., C. M. Small, K. A. Paczolt, and N. L. Ratterman. 2010. A practical guide to methods of parentage analysis. In Molecular Ecology Resources 10(1):6–30. 10.1111/j.1755-0998.2009.02778.x 21564987

[evl3250-bib-0044] Kalinowski, S. T., M. L. Taper, and T. C. Marshall. 2007. Revising how the computer program CERVUS accommodates genotyping error increases success in paternity assignment. Mol. Ecol. 16:1099–1106. 17305863 10.1111/j.1365-294X.2007.03089.x

[evl3250-bib-0045] Källander, H., and H. G. Smith. 1990. Manipulation of the brood size of pied flycatchers. Pp. 257–268 *in* J. Blondel, A. Gosler, J. D. Lebreton, and R. McCleery, eds. Population biology of passerine birds. Springer, Berlin.

[evl3250-bib-0046] Kemkes‐Grottenthaler, A. 2004. Parental effects on offspring longevity ‐ evidence from 17th to 19th century reproductive histories. Ann. Hum. Biol. 31:139–158. 15204358 10.1080/03014460410001663407

[evl3250-bib-0047] Kempenaers, B., and A. Dhondt. 1993. Why do females engage in extra‐pair copulations? A review of hypotheses and their predictions. Belgian J. Zool. 123:93–103.

[evl3250-bib-0048] Kempenaers, B., and B. C. Sheldon. 1996. Why do male birds not discriminate between their own and extra‐pair offspring? Anim. Behav. 51:1165–1173.

[evl3250-bib-0049] Kempenaers, B., G. R. Verheyen, M. Van Den Broeck, T. Burke, C. Van Broeckhoven, and A. Dhondt. 1992. Extra‐pair paternity results from female preference for high‐quality males in the blue tit. Nature 357:494–496.

[evl3250-bib-0050] Kiritani, K., and K. Kimura. 1967. Effects of parental age on the life cycle of the southern green stink bug, *Nezara viridula* L. (Heteroptera: Pentatomidae). Appl. Entomol. Zool. 2:69–78.

[evl3250-bib-0051] Knief, U., and W. Forstmeier. 2020. Violating the normality assumption may be the lesser of two evils. bioRxiv. 10.1101/498931 PMC8613103 33963496

[evl3250-bib-0052] Kokko, H. 1998. Good genes, old age and life‐history trade‐offs. Evol. Ecol. 12:739–750.

[evl3250-bib-0053] Kokko, H., and J. Lindstrom. 1996. Evolution of female preference for old mates. Proc. R. Soc. B Biol. Sci. 263:1533–1538.

[evl3250-bib-0054] Kong, A., M. L. Frigge, G. Masson, S. Besenbacher, P. Sulem, G. Magnusson, et al. 2012. Rate of de novo mutations and the importance of father's age to disease risk. Nature 488:471–475. 22914163 10.1038/nature11396 PMC3548427

[evl3250-bib-0055] Lansing, A. I. 1947. A transmissible, cumulative, and reversible factor in aging. J. Gerontol. 2:228–239. 20265000 10.1093/geronj/2.3.228

[evl3250-bib-0056] Lemaître, J. F., and J. M. Gaillard. 2017. Reproductive senescence: new perspectives in the wild. Biol. Rev. 92:2182–2199. 28374548 10.1111/brv.12328

[evl3250-bib-0057] Lifjeld, J. T., O. Kleven, F. Jacobsen, K. J. McGraw, R. J. Safran, and R. J. Robertson. 2011. Age before beauty? Relationships between fertilization success and age‐dependent ornaments in barn swallows. Behav. Ecol. Sociobiol. 65:1687–1697. 21949464 10.1007/s00265-011-1176-4 PMC3156913

[evl3250-bib-0058] Lind, M. I., E. C. Berg, G. Alavioon, and A. A. Maklakov. 2015. Evolution of differential maternal age effects on male and female offspring development and longevity. Funct. Ecol. 29:104–110.

[evl3250-bib-0059] Marshall, T. C., J. Slate, L. E. B. Kruuk, and J. M. Pemberton. 1998. Statistical confidence for likelihood‐based paternity inference in natural populations. Mol. Ecol. 7:639–655. 9633105 10.1046/j.1365-294x.1998.00374.x

[evl3250-bib-0060] Medawar, P. B. 1951. An unsolved problem of biology. H. K. Lewis & Co Ltd, Lond.

[evl3250-bib-0061] Michálková, R., O. Tomášek, M. Adámková, J. Kreisinger, and T. Albrecht. 2019. Extra‐pair paternity patterns in European barn swallows *Hirundo rustica* are best explained by male and female age rather than male ornamentation. Behav. Ecol. Sociobiol. 73:119.

[evl3250-bib-0062] Monaghan, P., A. A. Maklakov, and N. B. Metcalfe. 2020. Intergenerational transfer of ageing: parental age and offspring lifespan. Trends Ecol. Evol. 35:927–937. 32741650 10.1016/j.tree.2020.07.005

[evl3250-bib-0063] Newton, I., and P. Rothery. 2002. Age‐related trends in different aspects of the breeding performance of individual female Eurasian sparrowhawks (*Accipiter nisus*). Auk 119:735–748.

[evl3250-bib-0064] Noguera, J. C., N. B. Metcalfe, and P. Monaghan. 2018. Experimental demonstration that offspring fathered by old males have shorter telomeres and reduced lifespans. Proc. R. Soc. B Biol. Sci. 285 20180268. 10.1098/rspb.2018.0268 PMC5879639 29540524

[evl3250-bib-0065] Nussey, D. H., H. Froy, J. F. Lemaitre, J. M. Gaillard, and S. N. Austad. 2013. Senescence in natural populations of animals: widespread evidence and its implications for bio‐gerontology. Ageing Res. Rev. 12:214–225. 22884974 10.1016/j.arr.2012.07.004 PMC4246505

[evl3250-bib-0066] O'Brian, D. M. 1961. Effects of parental age on the life cycle of *Drosophila melanogaster* . Ann. Entomol. Soc. Am. 54:412–416.

[evl3250-bib-0067] Pizzari, T., R. Dean, A. Pacey, H. Moore, and M. B. Bonsall. 2008. The evolutionary ecology of pre‐ and post‐meiotic sperm senescence. Trends Ecol. Evol. 23:131–140. 18280006 10.1016/j.tree.2007.12.003

[evl3250-bib-0068] Preston, B. T., M. S. Jalme, Y. Hingrat, F. Lacroix, and G. Sorci. 2011. Sexually extravagant males age more rapidly. Ecol. Lett. 14:1017–1024. 21806745 10.1111/j.1461-0248.2011.01668.x

[evl3250-bib-0069] Priest, N. K., B. Mackowiak, and D. E. L. Promislow. 2002. The role of parental age effects on the evolution of aging. Evolution 56:927–935. 12093028 10.1111/j.0014-3820.2002.tb01405.x

[evl3250-bib-0070] Qvarnström, A., A. M. Rice, and H. Ellegren. 2010. Speciation in *Ficedula* flycatchers. Philos. Trans. R. Soc. B Biol. Sci. 365:1841–1852. 10.1098/rstb.2009.0306 PMC2871891 20439285

[evl3250-bib-0071] R Core Team . 2019. A language and environment for statistical computing. Available via https://www.r‐project.org/.

[evl3250-bib-0072] Rebke, M., T. Coulson, P. H. Becker, and J. W. Vaupel. 2010. Reproductive improvement and senescence in a long‐lived bird. Proc. Natl. Acad. Sci. USA 107:7841–7846. 20378836 10.1073/pnas.1002645107 PMC2867923

[evl3250-bib-0073] Rockstein, M. 1957. Longevity of male and female house flies. J. Gerontol. 12:253–256. 13463293 10.1093/geronj/12.3.253

[evl3250-bib-0074] Rodríguez‐Muñoz, R., P. Hopwood, D. Fisher, I. Skicko, R. Tucker, K. Woodcock, et al. 2019. Older males attract more females but get fewer matings in a wild field cricket. Anim. Behav. 153:1–14.

[evl3250-bib-0075] Sardell, R. J., P. Arcese, L. F. Keller, and J. M. Reid. 2011. Sex‐specific differential survival of extra‐pair and within‐pair offspring in song sparrows, *Melospiza melodia* . Proc. R. Soc. B Biol. Sci. 278:3251–3259. 10.1098/rspb.2011.0173 PMC3169025 21389032

[evl3250-bib-0076] Schroeder, J., S. Nakagawa, M. Rees, M. E. Mannarelli, and T. Burke. 2015. Reduced fitness in progeny from old parents in a natural population. Proc. Natl. Acad. Sci. USA 112:4021–4025. 25775600 10.1073/pnas.1422715112 PMC4386340

[evl3250-bib-0077] Shefferson, R. P., O. R. Jones, and R. Salguero‐Gómez. 2017. The evolution of senescence in the tree of life. Cambridge Univ. Press, Cambridge, U.K.

[evl3250-bib-0078] Sheldon, B. C. 1993. Sexually transmitted disease in birds: occurrence and evolutionary significance. Philos. Trans. R. Soc. London, B 339:491–497. 8098875 10.1098/rstb.1993.0044

[evl3250-bib-0079] Shirihai, H., and L. Svensson. 2018. *Handbook of Western palearctic birds, Volume 2*. Passerines: *flycatchers to buntings*. 1st ed. Bloomsbury Publishing, Lond.

[evl3250-bib-0080] Smeds, L., A. Qvarnström, and H. Ellegren. 2016. Direct estimate of the rate of germline mutation in a bird. Genome Res. 26:1211–1218. 27412854 10.1101/gr.204669.116 PMC5052036

[evl3250-bib-0081] Takagi, M. 2003. Different effects of age on reproductive performance in relation to breeding stage in Bull‐headed Shrikes. J. Ethol. 21:9–14.

[evl3250-bib-0082] Torres, R., H. Drummond, and A. Velando. 2011. Parental age and lifespan influence offspring recruitment: a long‐term study in a seabird. PLoS ONE 6 e27245. 22087271 10.1371/journal.pone.0027245 PMC3210767

[evl3250-bib-0083] Tracey, K. M. 1958. Effects of parental age on the life cycle of the mealworm, *Tenebrio molitor* Linnaeus. Ann. Entomol. Soc. Am. 51:429–432.

[evl3250-bib-0084] Trivers, R. L. 1972. Parental investment and sexual selection. Pp. 136–179 *in* B. Campbell, ed. Sexual selection and the descent of man, 1871–1971. Aldine, Chicago.

[evl3250-bib-0085] Velando, A., J. C. Noguera, H. Drummond, and R. Torres. 2011. Senescent males carry premutagenic lesions in sperm. J. Evol. Biol. 24:693–697. 21332857 10.1111/j.1420-9101.2010.02201.x

[evl3250-bib-0086] Weatherhead, P. J. 1984. Mate choice in avian polygyny: why do females prefer older males? Am. Nat. 123:873–875.

[evl3250-bib-0087] Westneat, D. F., and I. R. K. Stewart. 2003. Extra‐pair paternity in birds: causes, correlates, and conflict. Annu. Rev. Ecol. Evol. Syst. 34:365–396.

[evl3250-bib-0088] Wickham, H. 2016. ggplot2: elegant graphics for data analysis. Springer‐Verlag, New York. Available via https://ggplot2.tidyverse.org.

[evl3250-bib-0089] Zhang, H., O. Vedder, P. H. Becker, and S. Bouwhuis. 2015. Age‐dependent trait variation: the relative contribution of within‐individual change, selective appearance and disappearance in a long‐lived seabird. J. Anim. Ecol. 84:797–807. 25399484 10.1111/1365-2656.12321

