# Supplementary material for: Should females prefer old males?
Source: Evol Lett. 2021 Aug 24;5(5):507–20. doi: 10.1002/evl3.250 (PMC8484724; doi:10.1002/evl3.250)
Supplement: Supplementary file 1 — Figure S1. Age structure of the population since 2002 until 2018. Figure S2. Age structure of the population per years since (A) 2008 until (I) 2016. Table S1. A priori structure of the main models. Table S2. Age structure total known age individuals monitored between 2002 and 2018. Table S3. Linear mixed‐effect model (Gaussian) with fledgling number as response variable (cross‐sectional dataset). Table S4. Generalized mixed‐effect model (genpois) with fledgling number as response variable (cross‐sectional dataset). Table S5. Linear mixed‐effect model (Gaussian) with number of recruits as response variable (cross‐sectional dataset). Table S6. Generalized mixed‐effect model (genpois) with number of recruits as response variable (cross‐sectional dataset). Table S7. Linear mixed‐effect model with fledgling number as response variable (longitudinal dataset). Table S8. Generalized mixed‐effect model (genpois) with fledgling number as response variable (longitudinal dataset). Table S9. Linear mixed‐effect model (Gaussian) with number of recruits as response variable of longitudinal dataset (not including birds that died in year 1). Table S10. Generalized mixed‐effect model (genpois) with number of recruits as response variable of longitudinal dataset (not including birds that died in year 1). Table S11. Generalized mixed‐effect model (binomial) with recruitment as a response variable. Table S 12. Generalized mixed‐effect model with binomial distribution for the response variable Recruitment (yes, no) with male's age and male's survival as explanatory variables. Table S 13. Generalized mixed‐effect model with binomial distribution for the response variable Recruitment (yes, no) with male's age and male's survival as explanatory variables. [file EVL3-5-507-s001.docx]

**Title: Should females prefer old males?**

J. Carolina Segami^1†*^ – [carolina.segami@ebc.uu.se](mailto:carolina.segami@ebc.uu.se), +46728357394

Martin I. Lind^1†^ – [martin.lind@ebc.uu.se](mailto:martin.lind@ebc.uu.se)

Anna Qvarnström^1^ – [anna.qvarnstrom@ebc.uu.se](mailto:anna.qvarnstrom@ebc.uu.se)

^1^Department of Ecology and Genetics, Animal Ecology, Uppsala University, Norbyvagen 18D, 75236, Uppsala, Sweden.

†shared first authorship *corresponding author

Keywords: ageing, reproductive senescence, mate choice, female preference, germline senescence, direct benefits, genetic benefits, extra-pair mating

Type of article: Letter

**Supplementary tables and figures**

**
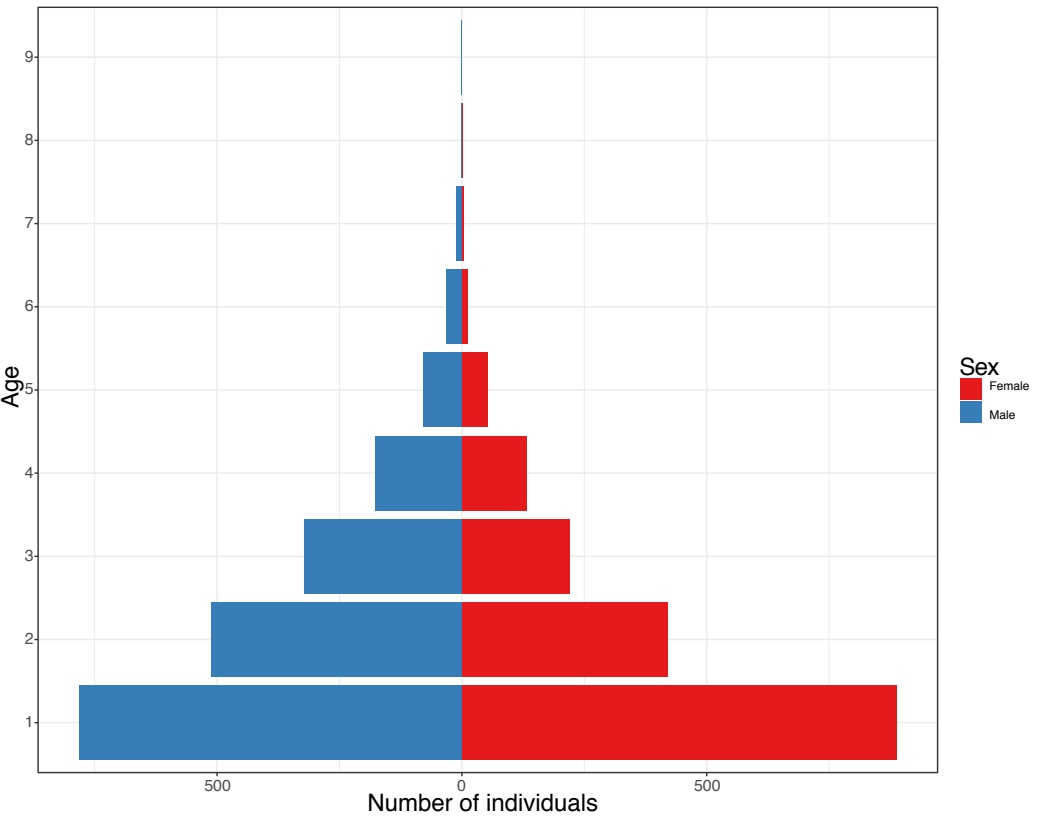
**

**Figure S1. Age structure of the population since 2002 until 2018.**


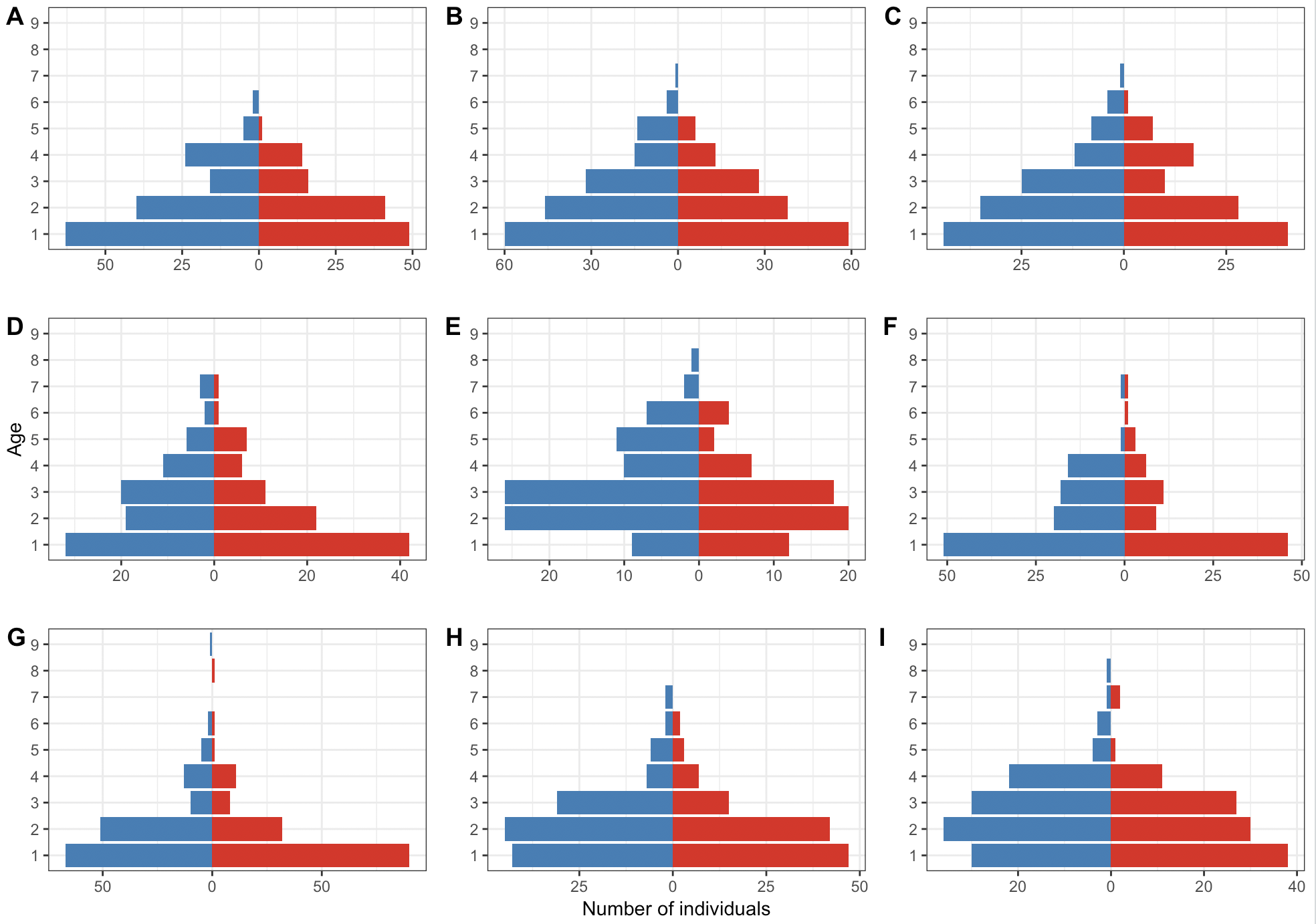
**Figure S2.** Age structure of the population per years since A. 2008 until I. 2016.

**Table S1.** A-priori structure of the main models. We use two datasets; (1) the long-term breeding dataset where we assess the combined direct and genetic effect of paternal age on offspring fledge number and recruitment, and (2) the extra-pair offspring dataset, where we disentangle direct and genetic effects of paternal age on offspring mass. Models using fledging and recruit numbers are analyzed both cross-sectional and longitudinally (excluding males not surviving to year 2). All models are analyzed in both general and generalized linear modeling framework. The results are robust to modeling framework.

| ***Long term breeding dataset*** | | | | | |
| --- | --- | --- | --- | --- | --- |
|  |  |  |  |  |  |
|  |  | *Cross-sectional* | | *Longitudinal* | |
| Response | Predictors | General | Generalized | General | Generalized |
| Fledging number | age + age^2^ | Table S3 | Table S4 | Table S7 | Table S8 |
| Recruit number | age + age^2^ | Table S5 | Table S6 | Table S9 | Table S10 |
| Recruit probability | age + age^2^  + survived  + age × survived + age^2^ × survived | - | - | - | Table S12, S13 |
|  |  |  |  |  |  |
| ***Extra-pair offspring dataset*** | | | | | |
|  |  |  |  |  |  |
| Response | Predictors |  |  | General | Generalized |
| Offspring mass | age + age^2^ + EPY-status  + total offspring in nest  + age × EPY-status + age^2^ × EPY-status | | | Table 1 | - |

**Table S2.** Age structure total known age individuals monitored between 2002 – 2018. Total percentages and percentages divided by sex.

| **Age class** | **Total** | **Total females** | **Total males** | **Total Percentage** | **Female percentage** | **Male percentage** |
| --- | --- | --- | --- | --- | --- | --- |
| 1 | 1670 | 888 | 782 | 45.8% | 51.3% | 40.8% |
| 2 | 932 | 420 | 512 | 25.5% | 24.3% | 26.7% |
| 3 | 542 | 221 | 321 | 14.9% | 12.8% | 16.7% |
| 4 | 310 | 133 | 177 | 8.5% | 7.7% | 9.2% |
| 5 | 130 | 52 | 78 | 3.6% | 3% | 4.1% |
| 6 | 44 | 12 | 32 | 1.2% | 1.8% | 1.7% |
| 7 | 16 | 4 | 12 | 0.4% | 0.2% | 0.6% |
| 8 | 3 | 1 | 2 | 0.08% | 0.06% | 0.1% |
| 9 | 1 | - | 1 | 0.03% | 0% | 0.05% |
| Total | 3648 | 1731 | 1917 | 100% | 100% | 100% |
| 5 + | 194 | 69 | 125 | 5.3% | 4% | 6.5% |

**Table S3.** Linear mixed-effect model (Gaussian) with fledgling number as response variable (crossectional dataset). Age and age^2^ of the social male are the explanatory variables. Year and Male ID are random effects. Fitted with lme4. Number of observations: 1527.

|  | **Estimate** | **S.E.** | **t** | **p** | **CI 2.5% - 97.5%** |
| --- | --- | --- | --- | --- | --- |
| Intercept | 5.00 | 0.12 | 42.60 | **< 0.001** | **4.76 – 5.24** |
| Age | 0.25 | 0.06 | 4.51 | **< 0.001** | **0.14 - 0.36** |
| Age^2^ | -0.06 | 0.02 | -2.73 | **0.007** | **-0.10 - -0.02** |
|  |  |  | |  | |
| **Random effects** | **Σ** | **CI 2.5% - 97.5%** | | **N groups** | |
| Male ID | 0 | 0 – 0.55 | | 994 | |
| Year | 0.40 | 0.25 – 0.63 | | 17 | |
| Residual | 2.01 | 1.94 – 2.09 | | - | |

**Table S4.** Generalized mixed-effect model (genpois) with fledgling number as response variable (crossectional dataset). Age and age^2^ of the social male are the explanatory variables.

Year and Male ID are random effects. Fitted with glmmTMB Number of observations: 1527.

|  | **Estimate** | **S.E.** | **Z** | **p** | **CI 2.5% - 97.5%** |
| --- | --- | --- | --- | --- | --- |
| Intercept | 1.604 | 0.023 | 70.04 | **< 0.001** | **1.56 - 1.65** |
| Age | 0.049 | 0.011 | 4.39 | **< 0.001** | **0.03 - 0.07** |
| Age^2^ | -0.011 | 0.004 | -2.67 | **0.008** | **-0.02 - -0.003** |
|  |  |  | |  | |
| **Random effects** | **Σ** | **CI 2.5% - 97.5%** | | **N groups** | |
| Year | 0.077 | 0.05 - 0.12 | | 17 | |
| Male ID | 0.0 | 0 – Inf | | 994 | |

**Table S5.** Linear mixed-effect model (Gaussian) with number of recruits as response variable (crossectional dataset). Year and Male ID are random effects. Fitted with lme4. Number of observations: 1728.

|  | **Estimate** | **S.E.** | **t** | **p** | **CI 2.5% - 97.5%** |
| --- | --- | --- | --- | --- | --- |
| Intercept | 0.40 | 0.05 | 8.7 | **< 0.001** | **0.31 – 0.49** |
| Age | 0.06 | 0.02 | 3.53 | **< 0.001** | **0.03 – 0.09** |
| Age^2^ | -0.01 | 0.01 -1.86 | | 0.064 -0.03 **–** 0.001 | |
|  |  |  | |  | |
| **Random effects** | **Σ** | **CI 2.5% - 97.5%** | | **N groups** | |
| Year | 0.17 | 0.11 – 0.26 | | 17 | |
| Male ID | 0.16 | 0.06 – 0.23 | | 1094 | |
| Residual | 0.63 | 0.60 – 0.66 | | - | |

**Table S6.** Generalized mixed-effect model (genpois) with number of recruits as response variable (crossectional dataset). Year and Male ID are random effects. Fitted with glmmTMB. Number of observations: 1728.

|  | **Estimate** | **S.E.** | **Z** | **p** | **CI 2.5% - 97.5%** |
| --- | --- | --- | --- | --- | --- |
| Intercept | -1.16 | 0.18 | -6.296 | **< 0.001** | **-1.52 - -0.98** |
| Age | 0.18 | 0.05 | 3.648 | **< 0.001** | **0.08 - 0.28** |
| Age^2^ | -0.03 | 0.02 -1.841 | | 0.066 -0.07 - 0.00 | |
|  |  |  | |  | |
| **Random effects** | **Σ** | **CI 2.5% - 97.5%** | | **N groups** | |
| Year | 0.685 | 0.43 – 1.09 | | 17 | |
| Male ID | 0.395 | 0.23 – 0.67 | | 1094 | |

**Table S7.** Linear mixed-effect model with fledgling number as response variable (longitudinal dataset). Year is a random effect and Male ID was removed because it caused a singularity error. Fitted with lme4. Number of observations: 1147.

|  | **Estimate** | **S.E.** | **t** | **p** | **CI 2.5% - 97.5%** |
| --- | --- | --- | --- | --- | --- |
| Intercept | 5.07 | 0.12 | 40.94 | **< 0.001** | **4.81 – 5.32** |
| Age | 0.11 | 0.06 | 1.95 | 0.052 | -0.002 **–** 0.22 |
| Age^2^ | -0.03 | 0.02 | -1.35 | 0.178 | -0.08 **–** 0.01 |
|  |  |  |  |  |  |
| **Random effects** | **Σ** | **CI 2.5% - 97.5%** | | **N groups** | |
| Year | 0.40 | 0.24 – 0.65 | | 17 | |
| Residual | 1.97 | 1.89 – 2.05 | | - | |

**Table S8.** Generalized mixed-effect model (genpois) with fledgling number as response variable (longitudinal dataset). Year and Male ID are random effects. Fitted with glmmTMB. Number of observations: 1147.

|  | **Estimate** | **S.E.** | **Z** | **p** | **CI 2.5% - 97.5%** |
| --- | --- | --- | --- | --- | --- |
| Intercept | 1.62 | 0.02 | 71.11 | **< 0.001** | **1.58 - 1.66** |
| Age | 0.02 | 0.01 | 1.95 | 0.05 | -0.0001 - 0.042 |
| Age^2^ | -0.01 | 0.004 | -1.34 | 0.18 | -0.014 - 0.003 |
|  |  |  |  |  |  |
| **Random effects** | **Σ** | **CI 2.5% - 97.5%** | | **N groups** | |
| Year | 0.072 | 0.043 – 0.12 | | 17 | |
| Male ID | 0.00003 | 0 – Inf | | 617 | |

**Table S9.** Linear mixed-effect model (Gaussian) with number of recruits as response variable of longitudinal dataset (not including birds which died in year 1). Fitted with lme4. Number of observations: 1274.

|  | **Estimate** | **S.E.** | **t** | **p** | **CI 2.5% - 97.5%** |
| --- | --- | --- | --- | --- | --- |
| Intercept | 0.43 | 0.06 | 7.81 | **< 0.001** | **0.32 - 0.55** |
| Age | 0.04 | 0.02 | 2.06 | **0.04** | **0.001 - 0.08** |
| Age^2^ | -0.01 | 0.01 | -1.17 | 0.24 | -0.02 - 0.01 |
|  |  |  |  |  |  |
| **Random effects** | **Σ** | **CI 2.5% - 97.5%** | | **N groups** | |
| Year | 0.20 | **0.13 – 0.31** | | 17 | |
| Male ID | 0.17 | **0.05 – 0.24** | | 648 | |
| Residual | 0.66 | **0.63 – 0.69** | | - | |

**Table S10.** Generalized mixed-effect model (genpois) with number of recruits as response variable of longitudinal dataset (not including birds which died in year 1). glmmTMB. Number of observations: 1274.

|  | **Estimate** | **S.E.** | **Z** | **p** | **CI 2.5% - 97.5%** |
| --- | --- | --- | --- | --- | --- |
| Intercept | -1.08 | 0.19 | -5.62 | **< 0.001** | **-1.45 - -0.70** |
| Age | 0.11 | 0.05 | 2.21 | **0.03** | **0.01 - 0.21** |
| Age^2^ | -0.02 | 0.02 | -1.10 | 0.27 | -0.06 - 0.02 |
|  |  |  |  |  |  |
| **Random effects** | **Σ** | **CI 2.5% - 97.5%** | | **N groups** | |
| Year | 0.70 | **0.44 – 1.12** | | 17 | |
| Male ID | 0.39 | **0.22 – 0.68** | | 648 | |

**Table S11.** Generalized mixed-effect model (binomial) with recruitment as a response variable. To assess if mass at day 12 is a good proxy for recruitment. Number of observations: 21879.

|  | **Estimate** | **S.E.** | **Z** | **p** | **CI 2.5% - 97.5%** |
| --- | --- | --- | --- | --- | --- |
| Intercept | -6.093 | 0.481 | -12.681 | **< 0.001** | **-7.04 - -5.15** |
| Mass at day 12 | 0.268 | 0.026 | 10.116 | **< 0.001** | **0.22 - 0.32** |
| Lay date | -0.053 | 0.008 | -6.306 | **< 0.001** | **-0.07 - -0.04** |
|  |  |  | |  | |
| **Random effects** | **Σ** | **CI 2.5% - 97.5%** | | **N groups** | |
| Year | 0.883 | **0.57 - 1.37** | | 17 | |
| Nest ID | 0.691 | **0.58 - 0.82** | | 4384 | |
| Residual |  |  | | - | |

**Table S 12.** Generalized mixed-effect model with binomial distribution for the response variable Recruitment (yes, no) with male’s age and male’s survival as explanatory variables. Year and Nest ID were included as random effects. Number of observations: 1522.

|  | **Estimate** | **S.E.** | **Z** | **p** | **CI 2.5% - 97.5%** |
| --- | --- | --- | --- | --- | --- |
| Intercept | -2.79 | 0.17 | 16.16 | **< 0.001** | **-3.17 - -2.43** |
| Age | 0.13 | 0.05 | -2.49 | **0.013** | **0.02 - 0.23** |
| Age^2^ | -0.02 | 0.02 | 1.27 | 0.21 | -0.06 – 0.01 |
| survived | 0.21 | 0.1 | -2.18 | **0.03** | **-0.40 - -0.01** |
|  |  |  |  |  |  |
| **Random effects** | **σ** | **CI 2.5% - 97.5%** | | **N groups** | |
| Year | 0.62 | 0.39 - 1.04 | | 17 | |
| Male ID | 0.59 | 0.42 - 0.75 | | 989 | |

**Table S 13.** Generalized mixed-effect model with binomial distribution for the response variable Recruitment (yes, no) with male’s age and male’s survival as explanatory variables. Year and Nest ID were included as random effects. Number of observations: 1522.

|  | **Estimate** | **S.E.** | **Z** | **p** | **CI 2.5% - 97.5%** |
| --- | --- | --- | --- | --- | --- |
| Intercept | -2.75 | 0.18 | 15.48 | **< 0.001** | **-3.14 - -2.38** |
| Age | 0.14 | 0.07 | -2.04 | **0.042** | **0.002 - 0.28** |
| Survived | 0.15 | 0.12 | -1.28 | 0.20 | -0.09 - 0.39 |
| Age^2^ | -0.05 | 0.03 | 1.44 | 0.15 | -0.11- 0.02 |
| Age : Survived | -0.01 | 0.1 | 0.05 | 0.96 | -0.21 - 0.20 |
| Survived : Age^2^ | 0.03 | 0.04 | -0.79 | 0.43 | -0.05 - 0.11 |
|  |  |  |  |  |  |
| **Random effects** | **σ** | **CI 2.5% - 97.5%** | | **N groups** | |
| Year | 0.62 | 0.39 - 1.04 | | 17 | |
| Male ID | 0.59 | 0.42 - 0.75 | | 989 | |
